# Beyond one-size-fits-all: sensory regulation and age-specific design in pediatric healthcare environments

**DOI:** 10.3389/fped.2026.1790826

**Published:** 2026-07-08

**Authors:** Haripriya Sathyanarayanan, Luisa Caldas

**Affiliations:** 1 Department of Architecture, College of Environmental Design, University of California, Berkeley, CA, United States; 2 Berkeley XR Lab, University of California, Berkeley, CA, United States

**Keywords:** child hospitalized, emotional regulation, evidence-based practice, hospital design and construction, pediatric healthcare design, sensory gating, virtual reality

## Abstract

Pediatric healthcare design often applies generalized child-friendly principles across a broad age range, from neonates through adolescence, despite well-established developmental differences in sensory processing, autonomy needs, and emotional regulation. Visual elements such as artwork and visual openness (e.g., window or corridor exposure) are commonly assumed to function as positive distractions across pediatric settings. However, evidence supporting these assumptions across developmental stages remains limited. This paper proposes a shift from one-size-fits-all approaches toward age-sensitive, regulatory framing of art and visual design in pediatric healthcare environments. Drawing on literature from pediatric healthcare design, developmental psychology, and sensory modulation, we synthesize evidence suggesting that visual environments may support or undermine wellness depending on developmental stage, context, and cumulative sensory load. To situate this framework within an applied design context, we present empirical observations from an immersive virtual reality evaluation integrating eye-tracking and physiological sensing. Responses of younger children (8–11), older children (12–17), and parents were examined across variations in artwork, a large exterior window, a multi-feature visual condition, and a low-stimulation reference condition in pediatric patient rooms. Observed patterns indicate that visual features intended to promote comfort and engagement can have divergent regulatory responses across age groups, particularly in relation to perceived autonomy and visual complexity. We conclude by outlining implications for pediatric healthcare design that emphasize calibration rather than amplification of sensory input, and discuss how immersive, data-informed approaches can support developmentally responsive decisions about art and visual environments across pediatric settings.

## 1 Introduction

Visual design strategies in pediatric healthcare environments are often implemented as broadly child-friendly across a wide age range, from infancy through adolescence (1). However, children's sensory processing, autonomy needs, and regulation capacities change substantially across development, and these differences have direct implications for how visual environments are experienced during hospitalization (2).

Children and adolescents hospitalized in the United States for various clinical conditions typically experience hospital stays that average between 3.8 and 6.3 days across age groups (3). During hospitalization, pediatric patients and their families face significant stressors including invasive medical procedures, separation from familiar environments, and limited mobility. These stressors heighten anxiety and can negatively affect recovery in children, while caregivers experience elevated stress due to feelings of helplessness, emotional vulnerability, and limited agency (4, 5). The built environment plays an important role in either alleviating or exacerbating these stressors. Design features such as access to daylight and exterior views, spatial organization, and family accommodation have been associated with stress reduction and, in some contexts, improved recovery-related outcomes, although the strength and pediatric-specificity of evidence vary by feature and outcome (6, 7).

Current pediatric hospital design guidelines provide limited differentiation of visual and sensory strategies by age group. Recommendations for artwork, color, and visual openness are typically articulated at a high level, with minimal guidance on how these elements should be modulated across developmental stages or care contexts (8–10). The prevailing approach treats pediatrics as a single design category, implicitly grouping an 8-year-old recovering from appendicitis with a 16-year-old managing chronic illness.

A one-size-fits-all approach persists despite substantial evidence from developmental psychology that sensory processing, emotional regulation, and needs for privacy and autonomy change markedly from early childhood through adolescence (11). Younger children may derive reassurance from concrete, high-salience visual stimuli including familiar or playful imagery, particularly under stress. In contrast, adolescents often place greater value on autonomy, identity, and boundaries, and can be sensitive to environments perceived as infantilizing or limiting control (12, 13). These differences have direct implications for how children experience prolonged exposure to pediatric healthcare environments where they have limited agency over sensory input.

Visual design decisions are particularly vulnerable to this homogenizing tendency. Artwork, murals, and visually rich environments are commonly framed as positive distractions, presumed to have beneficial effects regardless of age or context (14). However, while the concept of positive distraction is well-established in adult healthcare design research, it is rarely linked to specific considerations of sensory load in pediatric settings (15). In pediatric care, where pain, uncertainty, and disrupted routines already elevate regulatory demand, additional visual complexity may function as cognitive or sensory burden rather than relief (16). In this paper, this is conceptualized as regulatory burden, defined as the cumulative cognitive, emotional, and sensory demands placed on individuals as they maintain physiological and attentional regulation within an environment, particularly under conditions of sustained exposure or limited control. This aligns with research in environmental psychology and human factors indicating that increased environmental complexity can elevate attentional demand and cognitive load (17), with implications for individuals already managing stressors.

Empirical studies of pediatric healthcare environments have largely relied on caregiver reports, retrospective surveys, or structured interventions such as art therapy or distraction during procedures to assess the pediatric experience and health outcomes (8, 18–20). While informative, these approaches offer limited insight into children's moment-to-moment responses to visual environments and do not capture physiological processes associated with emotional and behavioral regulation. Research addressing art in pediatric healthcare has therefore focused more on discrete activities than on the role of art as a continuous environmental condition (21). Findings from literature generally indicate short-term benefits for anxiety or pain reduction but are often context-specific and do not address how ongoing exposure to visual environments differentially affects children across age groups or developmental stages. To address these gaps, this paper presents a conceptual framework that positions sensory regulation as a design target in pediatric healthcare environments. The framework is supported by empirical observations from an immersive virtual reality-based evaluation of pediatric patient room design, previously reported in detail elsewhere (7), and reinterpreted here to examine how visual features may function as regulatory inputs across developmental groups. The aim is to clarify how art, visual complexity, and window conditions may differentially shape regulatory demand across age groups and to outline implications for age-specific pediatric healthcare design.

## 2 Conceptual framework: sensory regulation as a design target

Sensory regulation refers to the dynamic process through which individuals maintain an optimal level of physiological, emotional, and behavioral arousal in response to environmental stimuli, shaped through interactions between neurological processing and environmental context (2). In built environments, sensory inputs such as lighting conditions, spatial complexity, and visual features influence this regulatory balance. Healthcare settings often present complex sensory conditions that affect stress responses, coping capacity, and perceptions of care environments (15, 22).

For pediatric patients already experiencing stress, unfamiliarity, and reduced control, environmental design can mitigate sensory overload, helping avoid hyperarousal states that may hinder clinical procedures and amplify perception of pain and discomfort (16, 23, 24). Sensory processing and regulatory capacity also change across childhood and adolescence as cognitive and emotional systems develop. Younger children tend to rely more on environmental cues for emotional stability, while older children and adolescents increasingly seek autonomy and environments that support emerging independence (11, 12).

Research on sensory modulation environments, particularly in pediatric mental health and neurodevelopmental contexts, has emphasized the importance of adjusting sensory input to support regulation (23, 24). However, these insights have not been systematically translated into mainstream pediatric healthcare design, where visual enrichment is still frequently treated as inherently supportive rather than conditionally supportive.

Immersive evaluation methods provide a complementary approach to traditional design research by enabling controlled manipulation of environmental variables while capturing real-time physiological and visual engagement responses (7, 25). In this paper, empirical observations are drawn from an immersive virtual reality-based evaluation of pediatric patient room design previously reported in detail elsewhere (7). The present analysis uses this dataset selectively to support the proposed sensory regulation framework, focusing on visual features and age-related response patterns rather than reproducing the full experimental analysis. Although such methods do not replace *in situ* studies, they allow examination of design effects that are difficult to isolate in active clinical environments and offer access to non-verbal indicators of experience, particularly among younger children (26). Participants were community volunteers, not currently hospitalized children; findings therefore reflect responses to simulated environmental conditions rather than active inpatient stress states.

This paper advances a regulatory framing of visual design in pediatric healthcare, situating visual environments within a broader sensory system that influences emotional and physiological regulation. Within this perspective, the effects of visual design are understood as conditional and developmentally mediated rather than universally beneficial. The regulatory framework we propose emphasizes not just the presence of visual features, but their configuration and controllability. We present empirical observations to illustrate this framing from an immersive virtual reality evaluation integrating physiological sensing incorporating heart rate variability measures. The evaluation examines how younger children, older children, and parents respond to variations in artwork, visual complexity, and window conditions in pediatric patient rooms, illustrating patterns of divergence across age groups rather than establishing causal relationships.

We use sensory regulation as a heuristic for interpreting how visual environments may shift regulatory effort across developmental stages, rather than as a predictive or normative model. The framework conceptualizes visual features such as artwork, visual openness, and window views as inputs within a broader sensory system that place varying regulatory demands on pediatric patients. These effects are conditioned by developmental stage, perceived autonomy and control, cumulative sensory load, and duration of exposure, highlighting why similar visual features may function differently across pediatric age groups and care contexts. The discussion emphasizes calibration of visual input through placement, intensity, and controllability, rather than uniform visual enrichment. While our focus is pediatric patient rooms, the framework has broader relevance for design research concerned with sensory regulation across healthcare, educational, and care settings. Pediatric healthcare, characterized by heightened vulnerability and developmental diversity, provides a critical context to reconsider prevailing assumptions about visual design and advance more developmentally responsive, evidence-informed practice. Supportive design within this framework requires attention to cumulative sensory load across the entire patient room, rather than isolated consideration of individual visual features.

### 2.1 Sensory input, developmental stage, and the limits of positive distraction

Positive distraction-defined as environmental features that physiologically reduce stress by competing for attentional resources without requiring significant cognitive effort (14, 15)-has become healthcare design's standard rationale for pediatric artwork and visual enrichment. The logic seems sound: provide engaging environmental stimulus, shift children's attention away from stressors, reduce distress (27). Ulrich's foundational study on supportive design supports this principle for adults viewing nature. However, adult-derived supportive design concepts are frequently generalized to pediatric settings without clear evidence that the same visual strategies support regulation across pediatric developmental stages or across prolonged exposure.

Positive distraction is typically operationalized in pediatric design as a unidirectional benefit, more visual engagement equals better outcomes. This framing has limited attention to how the same stimulus imposes different regulatory demands across ages, or how cumulative exposure shifts a stimulus from engaging to burdensome over time. A brightly colored mural may provide momentary distraction during a brief procedure, but what about continuous exposure over a 5-day hospital stay? For which children? Under what circumstances?

Developmental research indicates that these questions are developmentally contingent. Children's attentional control, interpretation of meaning, and preferences for privacy and autonomy change substantially across middle childhood and adolescence (28, 29). Younger children respond to concrete, high-salience visual stimuli and derive reassurance from familiar or playful imagery, particularly under stress (30–32). Their cognitive processing benefits from clear, identifiable visual anchors in unfamiliar environments. In contrast, adolescents place greater value on cognitive and behavioral autonomy, privacy, identity, and boundaries. They are sensitive to environments perceived as infantilizing (representing developmental regression) or limiting control, including spaces saturated with imagery designed for younger children that undermine their self-image as emerging adults (12, 33).

These differences mean that visual enrichment cannot be assumed to be uniformly supportive across pediatric age groups. A visual environment must be treated as variable input with potential regulatory costs as well as benefits. In pediatric care, where pain, uncertainty, and disrupted routines already elevate regulatory demand, additional visual complexity can function as added cognitive or sensory burden rather than relief. This aligns with environmental psychology and human factors research indicating that environmental complexity alters attentional allocation and cognitive effort, particularly when individuals are already managing stressors or task demands.

The implications for design are clear: the question is not whether a visual feature is attractive or engaging in isolation, but whether it supports regulation within the child's broader load at a given moment. A colorful nature mural may provide positive distraction for an 8-year-old arriving anxious for surgery. The same mural may increase regulatory burden for a 15-year-old, spending their fifth consecutive day in the same room, already managing pain, social isolation, and loss of autonomy.

Current design practice rarely differentiates visual strategies by developmental stage. Generic child-friendly features are applied throughout pediatric units without systematic attention to how they function across ages or contexts. This reflects a broader gap: design guidelines emphasize what features to include but provide limited guidance on when, how, and for whom those features support regulation rather than imposing additional load (10).

### 2.2 Developmental shifts in autonomy, privacy, and control

Across middle childhood and adolescence, children's regulatory strategies and expectations of privacy and control change in ways that are directly relevant to inpatient environments (34, 35). Younger children often rely more heavily on caregiver proximity and environmental cues for reassurance during stress (36), whereas adolescents place greater salience on boundaries, self-presentation, and autonomy, and may experience imposed visibility or juvenile theming as misaligned with their developmental needs (12).

Privacy takes on particular salience during adolescence (33). Unlike younger children who may welcome caregiver presence and oversight, adolescents place greater value on boundaries and selective disclosure (37). Large observation windows designed for safety monitoring can conflict with adolescent needs for privacy and control over self-presentation. Similarly, fixed visual features such as wall-mounted artwork depicting cartoonish or juvenile themes, can undermine adolescents' sense of being treated with maturity and respect. This is not merely aesthetic preference. Research on adolescent development demonstrates that environments signaling respect for autonomy support coping and regulation, while those perceived as constraining autonomy increase stress (38).

The implication for design is straightforward: visual features that support connection and engagement for younger children can create regulatory burden for adolescents when they limit perceived control or impose infantilizing imagery (39). A mural of cartoon animals may comfort an 8-year-old arriving anxious for surgery. The same mural may increase distress for a 15-year-old managing chronic illness over repeated hospitalizations, reinforcing their loss of autonomy rather than supporting coping.

Current pediatric design practice rarely differentiates visual strategies by developmental stage. The same child-friendly features are applied throughout pediatric units, reflecting adult assumptions about what children need rather than evidence of how different age groups regulate during prolonged exposure to healthcare environments. This gap between design intent and developmental reality creates avoidable misalignment between environmental features and patient needs.

### 2.3 Sensory load and the problem of cumulative exposure

Most research on positive distraction examines brief exposure, viewing nature during a procedure, and engagement with artwork during a waiting room visit. These studies provide useful evidence that visual stimuli can shift attention and reduce momentary stress. However, brief distraction differs fundamentally from continuous exposure over multi-day hospitalizations. During this time, children occupy patient rooms where visual features are fixed and unavoidable. Many pediatric patients, particularly those under intensive monitoring or with mobility restrictions, spend the majority of their stay within a single room. Visual features intended to engage or comfort during brief encounters become part of the continuous sensory environment (40).

Environmental psychology and human factors research demonstrate that the same stimulus can shift from supportive to burdensome depending on exposure duration and controllability (41). High-salience visual features that capture attention are effective for brief distraction precisely because they are salient (17). However, salience demands attentional resources. When exposure is continuous and cannot be modulated by the viewer, high-salience features can impose sustained vigilance rather than providing relief. This effect compounds when multiple visual features are layered within the same space. A pediatric patient room may contain wall-mounted artwork, decorative ceiling tiles, window views, privacy curtains with patterned designs, medical equipment with visual displays, and family photographs or personal items. Each feature is defensible in isolation. Collectively, they create a visually dense environment where children have limited ability to reduce input or shift focus away from visual stimuli.

For children already managing pain, anxiety, medication effects, or disrupted sleep, additional cognitive load from sustained visual processing can deplete regulatory resources. This is particularly relevant for older children and adolescents, who show greater sensitivity to environments perceived as visually overwhelming or lacking in opportunities for respite (40). Younger children may be more tolerant of high visual density when features are familiar or playful, but even for this age group, continuous exposure differs from brief engagement.

Supportive design requires attention to cumulative sensory load across the entire patient room rather than isolated consideration of individual features. Zoning strategies where some areas of the room provide high engagement and others provide low stimulation can support children's ability to modulate exposure. Features that can be activated or deactivated by patients or families (such as projection systems, adjustable lighting, or repositionable artwork) offer greater alignment with varying regulatory needs across the hospital stay.

Current design practice treats visual enrichment as universally supportive without systematic attention to cumulative effects or controllability. Guidelines recommend adding engaging features but provide limited direction on how to assess whether the total sensory environment exceeds children's capacity to regulate, particularly during prolonged exposure. This gap between brief distraction evidence and continuous exposure reality creates environments that may inadvertently increase regulatory demand for the children they are meant to support.

### 2.4 Regulatory mechanisms: how visual environments influence physiological and emotional states

Understanding how visual features influence regulation requires attention to the mechanisms through which environmental input affects physiological and emotional states. Research in environmental psychology, neuroscience, and psychophysiology provides evidence that visual environments operate through multiple pathways.

Visual complexity and attentional demand represent one pathway. Environments with high visual complexity defined by density of features, contrast, and competing focal points require greater attentional resources to process. This increased processing load is commonly examined using eye-tracking metrics and autonomic indicators such as heart rate and related measures of physiological activation. For individuals who are relaxed and cognitively available, moderate visual complexity can be engaging. For individuals already under stress or managing multiple demands, the same complexity can function as additional load rather than engagement.

Access to nature and distant views represents a distinct pathway. Ulrich's stress reduction theory and Kaplan's attention restoration theory both posit that nature views support recovery from stress and attentional fatigue (42–44). The mechanisms proposed include fascination (effortless attention), sense of extent (perceptual distance and openness), and evolutionary predispositions toward natural settings. Empirical studies across healthcare and other settings demonstrate associations between nature exposure and reduced stress indicators including lower heart rate, faster recovery from stressors, and improved affect. These effects have been reported across diverse adult and mixed-age settings, but pediatric evidence remains more limited and feature-specific.

Perceived control and agency represent a third pathway particularly relevant to pediatric populations. Environmental features that afford choice, through adjustability, personalization, or selective engagement, support autonomy and reduce helplessness. This is consistent with self-determination theory, which posits that autonomy is a fundamental psychological need (45, 46). During hospitalization, children experience substantial loss of control over daily routines, social contact, and physical mobility. Environmental features that offer even modest opportunities for control can support coping by providing limited domains of agency. Conversely, features that are fixed, imposed, or perceived as limiting autonomy can reinforce helplessness and increase stress.

These pathways interact rather than operating independently. A visually complex environment with high attentional demand may be experienced positively when the individual has control over engagement (e.g., choosing to view artwork) but negatively when exposure is imposed. Similarly, nature views may support stress reduction in part because they provide perceptual relief from high-demand indoor environments, suggesting an interaction between visual complexity and restorative potential.

Developmental stage moderates these pathways. Younger children may derive greater benefit from high-salience, concrete visual features because such features support orientation and provide clear focal points during uncertainty. Differences in appraisal, meaning-making, and perceived agency can increase the salience of immediate environmental cues in shaping emotional state. Adolescents, with more developed cognitive capacities, are better able to mentally disengage from immediate environments but are simultaneously more sensitive to environments perceived as limiting autonomy or failing to respect their developmental stage.

The implication for pediatric healthcare design is that visual features should be understood as regulatory inputs with differential effects depending on complexity, controllability, exposure duration, and developmental stage. Rather than asking whether a feature is good or engaging, the relevant questions concern how a feature influences regulatory load across different moments of care and for different age groups. A nature view visible from bed may reduce physiological activation regardless of age by providing perceptual distance and restorative qualities. A wall-mounted mural with high visual complexity may engage younger children during brief exposure but increase regulatory demand for adolescents during continuous multi-day exposure, particularly when combined with other fixed visual features.

Current pediatric design guidelines provide limited attention to these mechanistic considerations. Features are selected based on assumptions about child preferences or child-friendly aesthetics without systematic assessment of how they influence regulatory processes. The result is environments designed with good intentions but insufficient attention to the underlying processes through which visual features affect children's physiological and emotional states during hospitalization.

**Figure 1:**
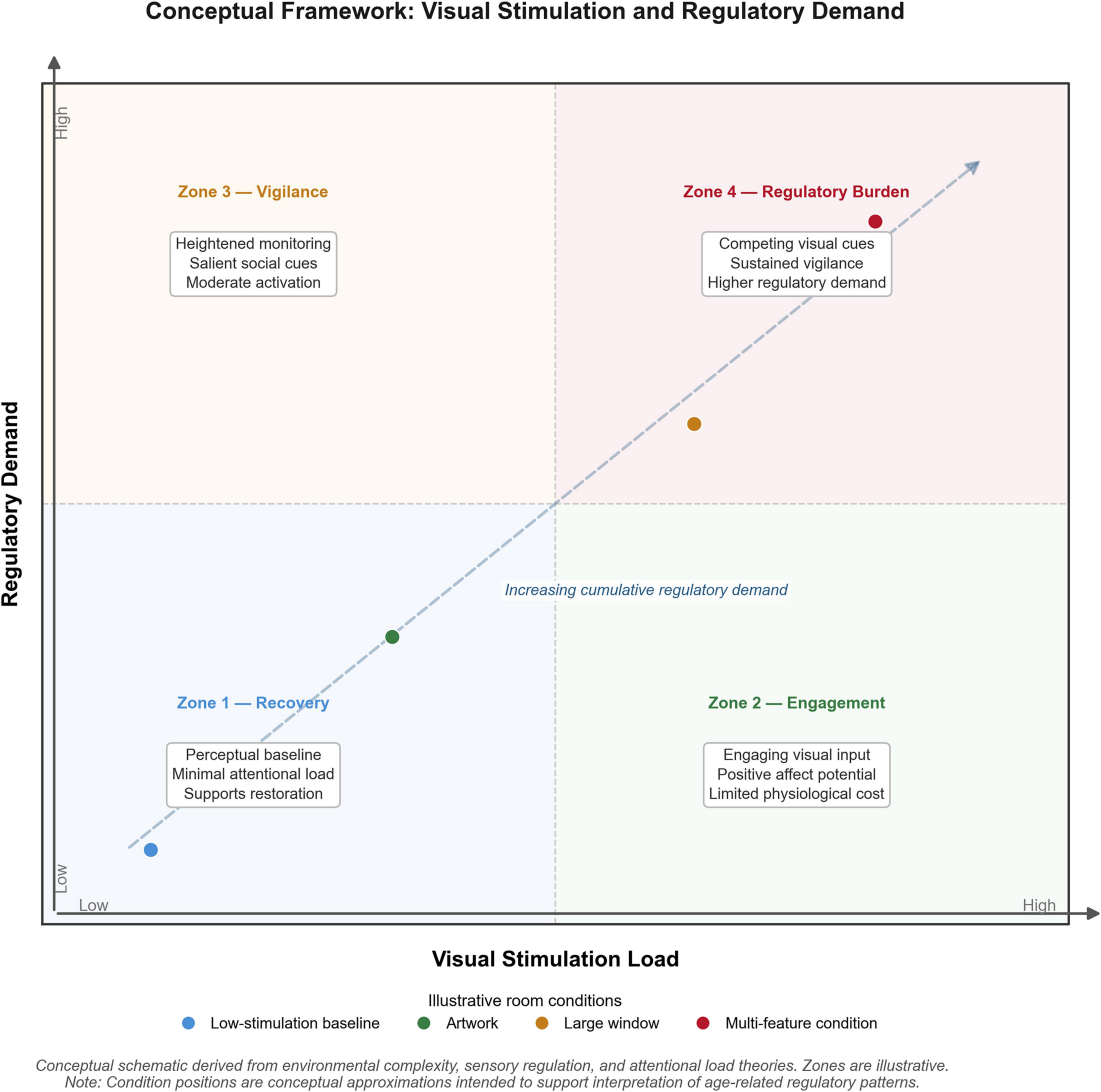
Conceptual framework relating visual stimulation load and regulatory demand in pediatric healthcare environments. The schematic synthesizes theory from environmental complexity, sensory regulation, attentional load, and developmental psychology to illustrate how increasing visual stimulation may impose variable regulatory demands depending on developmental stage, autonomy, and cumulative sensory exposure. The zones represent qualitative regulatory regimes rather than empirically estimated coordinates and are intended to support interpretation of age-related patterns observed in the valence–arousal distributions presented in subsequent analyses, rather than to predict specific outcomes or define optimal design thresholds.

Figure 1 summarizes this framework, emphasizing how visual features function as regulatory inputs whose effects are conditioned by developmental stage and exposure context.

## 3 Methods

### 3.1 Study design

This study presents data from an immersive virtual reality evaluation of pediatric patient room design, conducted to examine physiological, visual, and affective responses to controlled variations in room features. The experimental design, data collection procedures, and primary analyses are reported in detail elsewhere (7). The present paper draws selectively on this dataset to support a conceptual examination of visual design as a regulatory input, with attention to developmental differences across pediatric age groups. Methods are summarized to provide transparency sufficient for interpretation of the analyses and figures presented.

### 3.2 Participants

Twenty-nine individuals were recruited through convenience sampling via campus-wide listservs, parent networks, and the regional Children's Hospital. One participant who completed only one room layout was excluded, yielding a final sample of 28 participants who experienced both spatial layouts.

The sample comprised younger children ages 8–11 (*n* = 8, M = 9.3 years, SD = 1.2), older children ages 12–17 (*n* = 8, M = 14.6 years, SD = 1.9), and parents (*n* = 12, M = 42.8 years, SD = 9.1). See Table 1 for complete demographics. Among children, 7/16 (44%) reported prior hospitalization (≥3 days in pediatric unit within 5 years); all parents reported hospitalization experience (their own or their child's). Regarding VR familiarity, 15/28 (54%) reported no prior experience, 8/28 (29%) novice experience, and 5/28 (18%) occasional use.

**Table 1:** Participant demographics (*N* = 28).

Characteristic	Younger Children (8–11)	Older Children (12–17)	Parents	Total
N	8	8	12	28
Age (years)	M = 9.3, SD = 1.2	M = 14.6, SD = 1.9	M = 42.8, SD = 9.1	–
Gender				
Female	5 (62.5%)	3 (37.5%)	9 (75.0%)	17 (60.7%)
Male	3 (37.5%)	5 (62.5%)	3 (25.0%)	11 (39.3%)
Prior hospitalization				
Yes	2 (25.0%)	5 (62.5%)	12 (100%)	19 (67.9%)
No	6 (75.0%)	3 (37.5%)	0 (0%)	9 (32.1%)

Participants were screened via Qualtrics for conditions contraindicating VR use (motion sickness, vertigo, epilepsy, neurological disease). Those with prescription glasses were excluded if unable to participate without spectacles due to headset fit constraints. Depth perception was assessed through successful calibration of eye-tracking during the orientation protocol. Neurodevelopmental status (e.g., autism spectrum disorder, ADHD) was not formally assessed during screening. Age grouping (8–11 vs. 12–17) aligned with Piagetian developmental stages to examine differences in autonomy needs and sensory processing. Children younger than eight years were not included due to technical constraints associated with the VR and biosensing equipment. The HTC Vive Pro Eye headset and integrated EmteqPro sensors require stable headset positioning and facial contact to ensure reliable eye-tracking and facial electromyography (fEMG) measurements. Pilot testing indicated that the headset could not be reliably fitted on younger children due to head size and facial geometry, which can compromise calibration accuracy and sensor signal quality. The protocol requirements for sustained attention, and the study's focus on developmental differences most salient in middle childhood through adolescence.

All procedures were approved by the Institutional Review Board (Protocol #2022-02-15066). Parents provided informed consent; children provided assent.

### 3.3 Virtual environment and experimental conditions

The immersive environment consisted of a full-scale pediatric inpatient room modeled using industry-standard architectural workflows and experienced through a HTC Vive Pro Eye head-mounted display with integrated eye tracking and facial electromyography (fEMG) sensors. The base room geometry was derived from contemporary single-patient pediatric inpatient layouts and included a patient bed, family zone, clinical equipment, storage, and a window wall.

Two spatial layouts were evaluated: an outboard configuration, in which the patient room was oriented toward an interior corridor condition, and an inboard configuration, in which the room was oriented toward an exterior façade with access to daylight and views. Layouts differed in window placement and orientation while maintaining consistent room size, furniture arrangement, and clinical equipment. Within each layout, multiple visual room conditions were evaluated. The full study included seven room variants per layout. For the present paper, analyses focus on four representative conditions that capture a range of visual stimulation while preserving conceptual clarity:

1. Low-stimulation reference condition, characterized by minimal artwork and reduced visual complexity.
2. Artwork condition, featuring wall and ceiling artwork without modifications to window size or corridor visibility.
3. Large-window condition, emphasizing expanded exterior views and daylight access.
4. High-stimulation multi-feature condition, combining multiple visually salient elements, including artwork, increased visual complexity, and enhanced window features.

Room geometry, lighting levels, color, textures, and furniture were held constant across conditions, with only visual features manipulated. Each participant experienced multiple room conditions in randomized order within each layout. Exposure duration was standardized across conditions. Participants were instructed to explore the room naturally without task-based demands. Short rest periods were provided between exposures to minimize carryover effects. Individual room exposures lasted approximately 2.5–3 min, with total session duration approximately 30 min.

### 3.4 Measures

Physiological measures included heart rate and heart rate variability metrics commonly used to index autonomic activation and regulation. Facial electromyography was used to derive affective valence proxies based on zygomatic and corrugator muscle activity. Eye-tracking measures captured visual engagement patterns across room surfaces and features. Subjective responses were collected after each exposure using brief, semi structured interview. In the present paper, subjective measures are referenced only for contextual interpretation. Physiological and affective measures form the primary basis for the illustrative analyses presented.

### 3.5 Data processing and analysis

For the present analysis, physiological and affective measures were aggregated at the participant level and then summarized by group, layout, and room condition. Valence was represented using a normalized proxy derived from facial electromyography signals and scaled to a −1 to +1 range for visualization. Arousal was represented using a normalized activation proxy derived from heart-rate–based measures, clipped at zero to reflect relative increases in activation. Group-level median centroids were computed for each combination of participant group, room condition, and layout. Median aggregation was selected to reduce sensitivity to skewed distributions and inter-individual variability. The resulting centroids were visualized within a valence–arousal circumplex to support qualitative comparison across age groups, layouts, and visual conditions. These analyses are descriptive and intended to illustrate relative patterns of regulatory demand rather than to establish statistical significance or predictive thresholds.

The study protocol was reviewed and approved by the relevant institutional review board. All procedures complied with institutional ethical standards for research involving human participants.

### 3.6 Illustrative evidence from immersive evaluation

The regulatory framework proposed raises a practical question: do visual design features commonly assumed to support pediatric wellness function similarly across age groups when experienced as part of a patient room? To examine this question, we draw illustratively on empirical observations from an immersive evaluation integrating virtual reality, eye-tracking, and physiological sensing, described in detail in prior methodological reporting (7).

The immersive environment represented a pediatric patient room in which visual features could be introduced or removed while holding other spatial variables constant. Four conditions were examined: a room with wall-mounted artwork, a room with a large exterior window, a multi-feature room combining several visually salient elements, and a low-stimulation reference condition. Participants included younger children (ages 8–11), older children (ages 12–17), and parents, allowing comparison across developmental stages as well as between pediatric users and adult caregivers. Physiological measures provided relative indicators of autonomic activation, while facial electromyography and eye-tracking offered complementary information about affective valence and visual engagement.

#### 3.6.1 Valence and arousal patterns across age groups and room conditions

Table 2 presents mean valence and arousal values by participant group, room layout, and environmental condition. Valence and arousal were derived from facial electromyography and heart rate variability measurements, respectively, with values calculated as participant-level means across the full duration of each room exposure.

**Table 2:** Mean valence and arousal by group, layout, and room condition.

Group	Layout	Condition	Valence M (SD)	Arousal M (SD)	*n*
Younger Child	Inboard	Low-stimulation (baseline)	−0.11 (0.26)	0.19 (0.38)	7
		Artwork	−0.15 (0.33)	0.22 (0.26)	8
		Large window	0.06 (0.26)	0.15 (0.11)	8
		High-stimulation (multi-feature)	0.05 (0.45)	0.19 (0.21)	7
	Outboard	Low-stimulation (baseline)	0.09 (0.43)	0.18 (0.21)	6
		Artwork	0.00 (0.34)	0.24 (0.30)	5
		Large window	0.10 (0.42)	0.14 (0.21)	5
		High-stimulation (multi-feature)	0.01 (0.52)	0.24 (0.26)	5
Older Child	Inboard	Low-stimulation (baseline)	−0.14 (0.51)	0.13 (0.21)	7
		Artwork	−0.08 (0.38)	0.26 (0.27)	7
		Large window	0.01 (0.40)	0.19 (0.26)	8
		High-stimulation (multi-feature)	0.04 (0.36)	0.36 (0.32)	7
	Outboard	Low-stimulation (baseline)	0.05 (0.32)	0.19 (0.28)	6
		Artwork	−0.09 (0.47)	0.24 (0.36)	5
		Large window	0.06 (0.11)	0.05 (0.10)	5
		High-stimulation (multi-feature)	0.22 (0.40)	0.25 (0.41)	4
Parent	Inboard	Low-stimulation (baseline)	−0.23 (0.32)	0.03 (0.13)	5
		Artwork	0.29 (0.42)	0.11 (0.31)	7
		Large window	0.23 (0.32)	0.02 (0.16)	8
		High-stimulation (multi-feature)	−0.31 (0.40)	0.11 (0.18)	6
	Outboard	Low-stimulation (baseline)	−0.01 (0.26)	0.02 (0.06)	7
		Artwork	−0.18 (0.39)	0.14 (0.14)	6
		Large window	0.25 (0.32)	0.13 (0.23)	6
		High-stimulation (multi-feature)	0.33 (0.30)	0.14 (0.50)	6

Valence scaled from −1 (negative) to +1 (positive); Arousal scaled from 0 (low) to 1 (high). Values represent participant-level means aggregated by condition.

##### 3.6.1.1 Younger children (ages 8–11)

In the inboard layout, younger children showed mean valence of −0.15 (SD = 0.33) for artwork, 0.05 (SD = 0.45) for high-stimulation, −0.11 (SD = 0.26) for low-stimulation baseline, and 0.06 (SD = 0.26) for large window conditions. Mean arousal was 0.22 (SD = 0.26) for artwork, 0.19 (SD = 0.21) for high-stimulation, 0.19 (SD = 0.38) for baseline, and 0.15 (SD = 0.11) for window conditions.

In the outboard layout, younger children exhibited mean valence of 0.00 (SD = 0.34) for artwork, 0.01 (SD = 0.52) for high-stimulation, 0.09 (SD = 0.43) for baseline, and 0.10 (SD = 0.42) for window conditions. Mean arousal was 0.24 (SD = 0.30) for artwork, 0.24 (SD = 0.26) for high-stimulation, 0.18 (SD = 0.21) for baseline, and 0.14 (SD = 0.21) for window conditions.

##### 3.6.1.2 Older children (ages 12–17)

In the inboard layout, older children showed mean valence of −0.08 (SD = 0.38) for artwork, 0.04 (SD = 0.36) for high-stimulation, −0.14 (SD = 0.51) for baseline, and 0.01 (SD = 0.40) for window conditions. Mean arousal was notably higher for high-stimulation (0.36, SD = 0.32) and artwork (0.26, SD = 0.27) conditions compared to baseline (0.13, SD = 0.21) and window (0.19, SD = 0.26) conditions.

In the outboard layout, older children exhibited mean valence of −0.09 (SD = 0.47) for artwork, 0.22 (SD = 0.40) for high-stimulation, 0.05 (SD = 0.32) for baseline, and 0.06 (SD = 0.11) for window conditions. The large window condition showed notably low arousal (0.05, SD = 0.10) relative to other conditions.

##### 3.6.1.3 Parents

In the inboard layout, parents showed mean valence of 0.29 (SD = 0.42) for artwork, −0.31 (SD = 0.40) for high-stimulation, −0.23 (SD = 0.32) for baseline, and 0.23 (SD = 0.32) for window conditions. Mean arousal was low across all conditions, ranging from 0.02 (SD = 0.16) for window to 0.11 (SD = 0.18–0.31) for other conditions.

In the outboard layout, parents exhibited mean valence of −0.18 (SD = 0.39) for artwork, 0.33 (SD = 0.30) for high-stimulation, −0.01 (SD = 0.26) for baseline, and 0.25 (SD = 0.32) for window conditions. Arousal values ranged from 0.02 (SD = 0.06) for baseline, 0.13 (SD = 0.23) for large window, to 0.14 (SD = 0.14) for artwork and 0.14 (SD = 0.50) for high-stimulation conditions.

**Figure 2:**
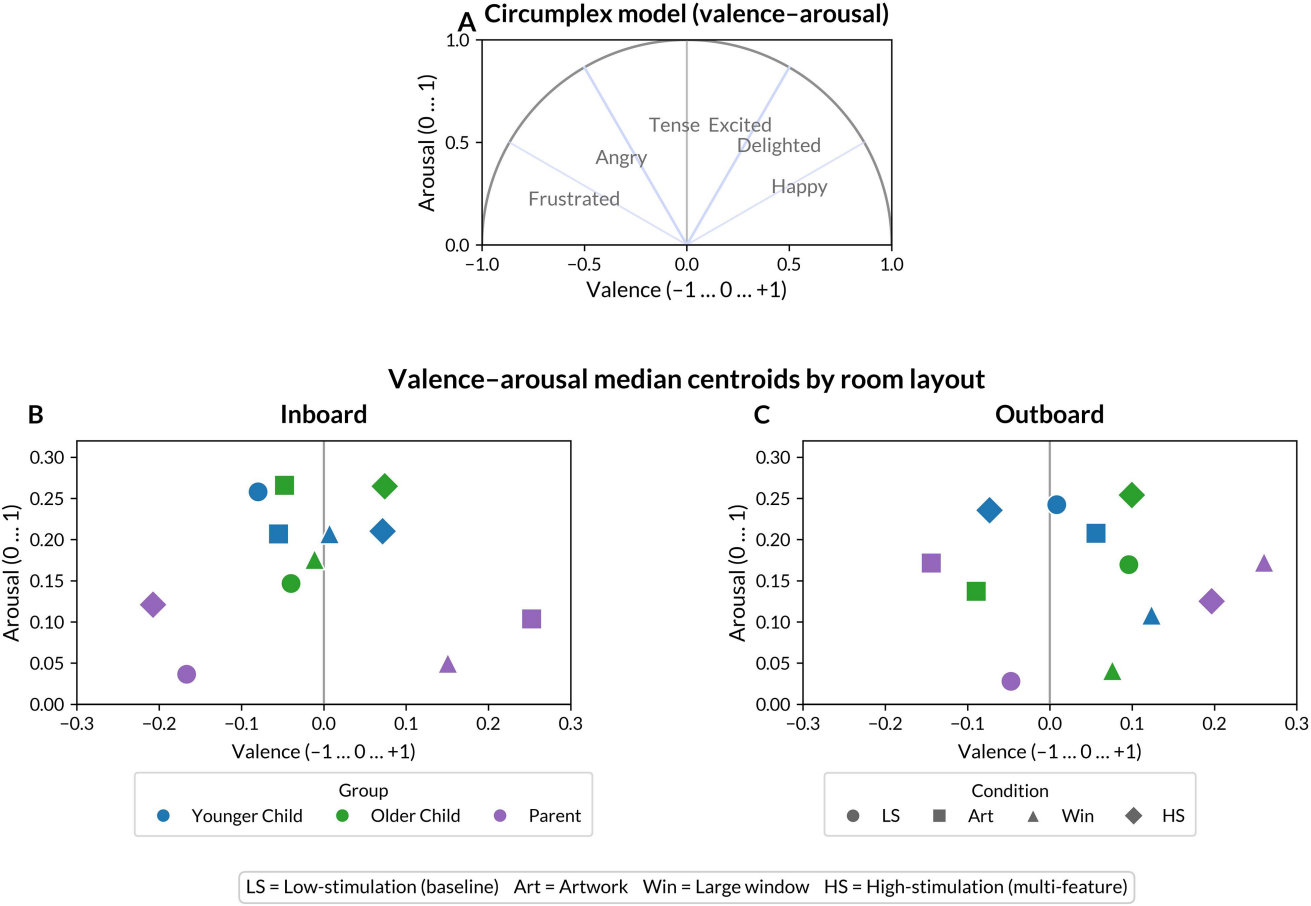
Valence–arousal circumplex model and median centroids by room layout. **(A)** Conceptual valence–arousal circumplex illustrating qualitative affective regions (e.g., frustrated, angry, tense, excited, delighted, happy). Valence is shown on the horizontal axis (−1 to +1), and arousal on the vertical axis (0 to 1). Radial guides indicate approximate affective sectors and are provided for conceptual reference only. **(B)** Median valence–arousal centroids for the inboard room layout. **(C)** Median valence–arousal centroids for the outboard room layout. In panels **(B)** and **(C)**, points represent group-level median centroids derived from aggregated responses. Color indicates participant group (Younger Child, Older Child, Parent), and marker shape indicates room condition: low-stimulation baseline (LS), artwork (Art), large window (Win), and high-stimulation multi-feature condition (HS). Valence represents a normalized proxy scaled to the range −1 to +1, and arousal represents a normalized activation proxy scaled from 0 to 1. Axes are held constant across layouts to support visual comparison.

Figure 2 visualizes median centroids for each group and condition within a valence–arousal circumplex. The low-stimulation reference condition consistently clusters near neutral valence and lower arousal across both inboard and outboard layouts, functioning as a regulatory baseline rather than a deficit condition. Artwork-only conditions show modest positive shifts in valence with relatively limited changes in arousal, indicating affective engagement without substantial activation. In contrast, conditions emphasizing visual access to the exterior extend further along the arousal dimension while remaining within the positive valence range. The high-stimulation multi-feature condition occupies a distinct region characterized by elevated arousal and greater separation across participant groups, reflecting more variable regulatory responses. While the relative ordering of conditions is similar across layouts, arousal-related separation is more pronounced in the outboard configuration, indicating that spatial layout mediates how visually salient features are experienced. These findings are patterns that demonstrate how regulatory responses to visual environments can diverge systematically across age groups under controlled conditions.

#### 3.6.2 Qualitative responses to room features

Following immersive exposure, participants provided verbal responses regarding preferred and disliked features across room conditions. Thematic patterns emerged that varied by age group and room type.

Younger children consistently emphasized visual richness and color. Descriptions included references to “colors on walls,” “patterns and cushions,” “art with colors and lot of different things to see,” and “lots to look at.” Several younger participants associated artwork with emotional safety, with one stating “art makes me feel safer.” Preferences centered on abundance of visual input rather than specific content, with the multi-feature condition frequently described as “amazing” or “favorite.” Privacy concerns related to the visible corridor were mentioned but typically framed as discomfort with openness (“don’t know why I did not like it”) rather than explicit autonomy language.

Older children articulated different priorities. While some appreciated visual features (“ceiling art was nice”), others described feeling “bored after a point” or found pediatric imagery “childish.” Privacy emerged as a central concern, with the nurse view window described as causing “intrusion of privacy,” “intimidating,” or “overwhelming.” The visible corridor elicited stronger reactions: “very open, no privacy,” “felt like a subway station,” “very intimidating.” Multiple older participants requested control mechanisms (“privacy curtain,” “Want control over lighting, temperature”). Responses to the multi-feature condition ranged from “comfortable and not professional” to “over stimulating,” with appreciation expressed when layered features signaled age-appropriate rather than institutional design.

Parent responses reflected dual concerns for connection and privacy. Some valued visibility features for caregiving purposes (“I want either the nurse window or the visible corridor… can feel isolating”), while others expressed privacy concerns mirroring older children's perspectives (“nurse window was too prying,” “visible corridor uncomfortable”). Preferences for warmth and connection to the environment were common, with references to “warm yellow colors,” “wood,” and “plants” as contributing to comfort. Spatial considerations related to caregiver positioning and family accommodation were frequently mentioned.

Window features received consistently positive descriptions across all age groups. Comments included “larger the windows the better,” “room felt large and spacious with the large windows,” “without window you feel stuck in the space,” and “windows… want healing and relaxing feeling.” No participant expressed preference for smaller windows or absence of exterior views, though practical considerations regarding climate control were occasionally noted.

## 4 Discussion: implications for pediatric art and visual design

The patterns observed in this evaluation suggest visual environments in pediatric healthcare may function as regulatory inputs with age-mediated effects rather than as uniformly supportive features. These preliminary findings align with established research on developmental differences in sensory processing, autonomy needs, and environmental preferences while raising questions about prevailing assumptions in pediatric design practice.

Visual design in pediatric healthcare has historically relied on adult-derived principles of positive distraction and supportive design (15, 27). Ulrich's foundational work demonstrated that nature views reduce stress in hospitalized adults through mechanisms including distraction from worrisome thoughts and physiological restoration (42). Attention restoration theory extends this framework, proposing that natural environments support recovery from directed attention fatigue through effortless engagement (43). These principles have been generalized to pediatric settings with limited attention to developmental differences in how visual environments are processed and experienced. Research on children's environmental preferences demonstrates that aesthetic judgments and responses to visual complexity change substantially across childhood and adolescence (39), yet pediatric design guidelines typically provide minimal differentiation of strategies by developmental stage (8–10).

The divergent responses to visual features observed across age groups align with research on developmental shifts in autonomy, privacy, and self-presentation during adolescence. Adolescents place greater emphasis on environments that support emerging independence and respect developmental maturity compared to younger children (12, 33, 37). This shift has direct implications for healthcare environments, where perceived control over the physical environment can support coping during hospitalization (13, 45). The emphasis on privacy and control expressed by older participants in this study mirrors findings from research on adolescent healthcare experiences, which indicates that environments perceived as infantilizing or limiting autonomy may undermine rather than support regulation during this developmental period (12). In contrast, younger children's regulatory patterns may be more heavily influenced by caregiver presence and familiar environmental cues (36), consistent with their emphasis on visual richness and safety-related associations with artwork in this evaluation.

The observed patterns related to cumulative sensory load reflect broader evidence on environmental complexity and attentional demand. Research in environmental psychology demonstrates that visual complexity influences cognitive load and attentional allocation, with effects moderated by individual stress levels and task demands (17). For individuals managing stressors, high-complexity environments may impose sustained attentional costs rather than providing respite. This dynamic is particularly relevant in pediatric healthcare settings, where children face pain, medical procedures, and disrupted routines that already elevate regulatory demand. Evidence from sensory modulation environments, primarily developed in pediatric mental health and neurodevelopmental contexts, demonstrates that calibrating sensory input to individual capacity supports emotional and behavioral regulation (23, 24). These principles have not been systematically translated into mainstream pediatric healthcare design, where visual enrichment is typically treated as inherently beneficial rather than conditionally supportive depending on cumulative load and controllability.

The relatively consistent patterns observed for window conditions across age groups align with evidence on biophilic design and access to nature in healthcare settings. Studies across diverse adult populations demonstrate associations between window access and reduced pain medication use, shorter hospital stays, and improved recovery outcomes (6). The proposed mechanisms include restoration of directed attention capacity, reduction in physiological stress markers, and provision of temporal variation that supports circadian regulation. Pediatric evidence on window access remains more limited, though environmental preference studies indicate children value connection to exterior environments (31, 32, 44). The low arousal patterns observed across age groups for window conditions in this evaluation, combined with consistently positive qualitative responses, suggest that windows may support regulation through mechanisms distinct from artwork or designed features. Nature views provide perceptual depth and temporal variation without requiring sustained cognitive engagement with fixed visual content, potentially explaining their broader appeal across developmental stages.

Translation of these preliminary observations to practice requires attention to several methodological and contextual factors. Participants were not undergoing active hospitalization; therefore, the observed responses reflect environmental perception and regulatory processing under controlled conditions rather than the full physiological and psychological states associated with inpatient care, including pain, medical uncertainty, disrupted routines, and sustained loss of control. Adaptation patterns across extended hospitalization, interactions between environmental features and clinical variables, and individual differences in sensory processing thresholds remain to be systematically examined. The substantial within-group variability observed in physiological measures underscores individual differences in regulatory responses that are not captured by age-based categorization alone. Children with neurodevelopmental differences, chronic conditions affecting sensory processing, or prior trauma exposure may exhibit regulatory patterns that diverge from those observed in typically developing samples.

Current design practice emphasizes addition of engaging features without systematic frameworks for assessing cumulative sensory load or supporting patient control over environmental exposure. The regulatory framework proposed here suggests an alternative approach: calibration rather than amplification of visual input, with attention to developmental stage, exposure duration, and opportunities for modulation. Design strategies that support this approach include zoning between high-stimulation and low-stimulation areas, features that can be activated or deactivated by patients and families, and prioritization of visual features with evidence of consistent regulatory support across developmental stages. As immersive technologies and physiological sensing become more accessible, integration of these methods into early-stage design evaluation offers opportunity for evidence-informed optimization before construction. However, such methods complement rather than replace *in situ* observation with hospitalized children across varied clinical and cultural contexts.

## 5 Future directions

The regulatory framework and preliminary observations presented here suggest several priorities for advancing this work.

In situ validation with hospitalized children represents the most critical next step. The patterns observed in this immersive evaluation require confirmation in actual pediatric inpatient settings where children experience illness-related stress and multi-day exposure. Longitudinal studies tracking responses across hospital stays would clarify whether reactions to visual features persist or change over time, ideally integrating patient-reported outcomes with physiological measures to examine relationships with clinical variables including pain, sleep quality, and recovery.

Extension to diverse populations is essential. Children with neurodevelopmental differences such as autism spectrum disorder or ADHD may exhibit distinct sensory processing thresholds requiring tailored design approaches. Children with chronic conditions requiring repeated hospitalization may develop different environmental preferences compared to those in acute care. Cultural context warrants investigation, as developmental norms for autonomy and privacy vary across populations.

Intervention studies testing specific design modifications would advance translation to practice. Controlled comparisons of zoning approaches, patient-controlled features, and calibrated visual complexity would clarify which strategies most effectively support regulation across age groups. Such studies could examine both physiological responses and longer-term outcomes including family satisfaction and clinical metrics.

Finally, mechanistic research integrating neuroimaging and biomarkers could elucidate pathways through which visual environments influence stress responses and emotional processing, informing more precise design recommendations.

## 6 Conclusion

Art and visual design in pediatric healthcare environments are often treated as universally supportive features intended to comfort, distract, or engage children during hospitalization. This paper challenges that assumption by reframing visual environments as components of sensory regulation rather than as uniformly positive additions. From this perspective, visual features impose regulatory demands that vary by developmental stage, autonomy needs, and cumulative exposure over time.

Drawing on theory from environmental psychology, developmental research, and sensory modulation, the paper advances a regulatory framework that emphasizes conditionality rather than generalization. The framework clarifies why similar visual strategies may support regulation for some pediatric users while increasing regulatory demand for others, particularly under conditions of prolonged exposure and limited control. Rather than evaluating visual features in isolation, the framework highlights the importance of considering how visual input is distributed, layered, and experienced across different moments of care.

Illustrative evidence from immersive evaluation situates this framework within an applied design context. Physiological measures and participant interviews converged in identifying developmental differences in regulatory responses, though with substantial individual variability within age groups. Observed patterns across younger children, older children, and parents suggest that visually engaging features such as artwork may support orientation and affective engagement for younger children, while visually dense or imposed environments may increase regulatory demand for older children and adolescents. In contrast, access to exterior views appears to operate more consistently across age groups, suggesting that not all forms of visual input function through the same regulatory pathways. These observations do not establish causal effects, but they underscore the importance of moving beyond one-size-fits-all assumptions in pediatric design.

The implication for practice is not to reduce visual richness across pediatric settings, but to calibrate visual environments to developmental and contextual needs. Supportive design emerges not from the presence of specific features, but from how visual input is modulated, controlled, and aligned with patients' regulatory capacity over time. Strategies that allow for variation, choice, and perceptual respite may better support coping during hospitalization than uniformly applied visual enrichment.

More broadly, this study demonstrates the value of integrating conceptual frameworks with immersive, data-informed evaluation to examine how pediatric environments are experienced. In settings where children face prolonged stress, limited agency, and developmental diversity, sensory regulation offers a useful lens for aligning design intent with lived experience. By treating visual design as a regulatory variable rather than a stylistic decision, pediatric healthcare design can move toward more developmentally responsive and evidence-informed practice.

## Data Availability

The raw data supporting the conclusions of this article will be made available by the authors, without undue reservation.

## Supplementary material

Supplementary Figure S1 Representative room conditions included in the sensory regulation framework. representative renderings and floor plans of the four conceptual room conditions evaluated in the study, shown for both the outboard and inboard layouts: low-stimulation, artwork, large-window, and high-stimulation.
