# Supplementary figures and images for: Beyond one-size-fits-all: sensory regulation and age-specific design in pediatric healthcare environments
Source: Front Pediatr. 2026 Jul 8;14:1790826. doi: 10.3389/fped.2026.1790826 (PMC13388379; doi:10.3389/fped.2026.1790826)

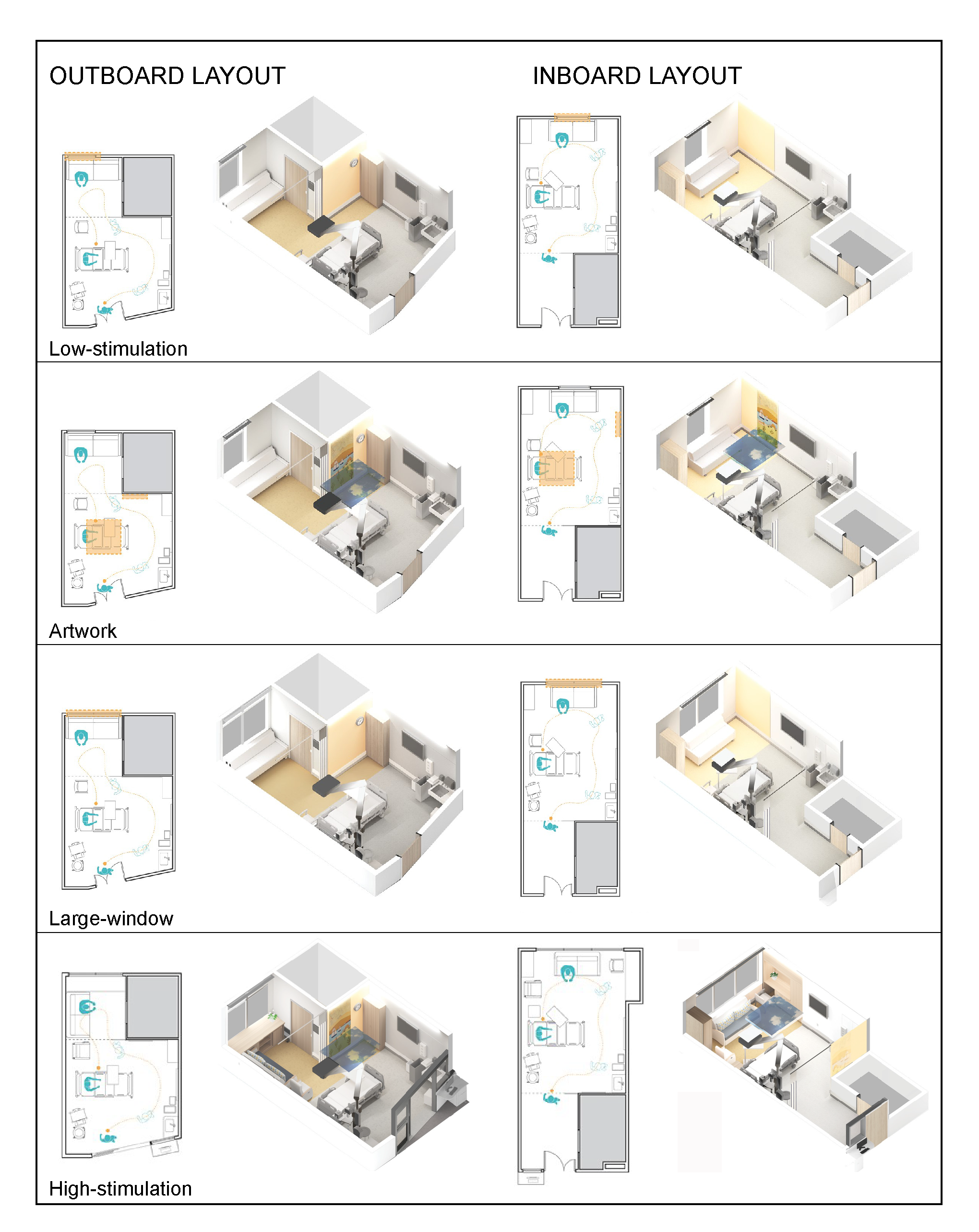

Supplement: Supplementary Figure S1 — Representative room conditions included in the sensory regulation framework. representative renderings and floor plans of the four conceptual room conditions evaluated in the study, shown for both the outboard and inboard layouts: low-stimulation, artwork, large-window, and high-stimulation. [file Image1.png]
